# Microbiota and Quality Indexes of Commercial Sauerkraut and Fermented Cucumbers

**DOI:** 10.1111/1758-2229.70250

**Published:** 2025-12-12

**Authors:** Renata Choińska, Katarzyna Piasecka‐Jóźwiak, Olga Świder, Agata Żak‐Kułakowicz, Karol Włodarczyk, Juliusz Załuski

**Affiliations:** ^1^ Department of Fermentation Technology Prof. W. Dąbrowski Institute of Agricultural and Food Biotechnology‐State Research Institute Warsaw Poland; ^2^ Department of Food Safety and Chemical Analysis Prof. W. Dąbrowski Institute of Agricultural and Food Biotechnology‐State Research Institute Warsaw Poland

**Keywords:** biogenic amines, fermented vegetables, lactobacilli, microbial analysis, nanopore sequencing

## Abstract

Sauerkraut and fermented cucumbers are the most commonly consumed fermented vegetables in several regions of Eastern, Central, and Western Europe; thus, their quality is of great importance. In this study, the quality and bacterial microbiota of commercial samples of fermented cucumbers and sauerkraut were assessed. The identification and quantification of the microbial consortia were performed using nanopore sequencing, and ultrahigh‐performance liquid chromatography–tandem mass spectrometry was used to analyse the content of biogenic amines. The physicochemical analysis showed great sample differentiation. Microbial analysis revealed a large diversity of types and relative abundances amongst the studied samples. *Lactobacillaceae* dominated the microbial community of fermented products, with the most common genera being *Latilactobacillus*, *Leuconostoc*, *Lactiplantibacillus*, *Levilactobacillus*, *Lentilactobacillus*, *Secundilactobacillus*, and *Pediococcus*. As for biogenic amines, putrescine prevailed both in the samples of fermented cucumber and sauerkraut, followed by cadaverine. The calculated biogenic amine index for four samples of sauerkraut and one of pickled cucumbers exceeded the estimated upper toxicity limit, ranging from 392 to 541, respectively. Spearman correlation analysis showed a significant positive correlation between *Secundilactobacillus* and cadaverine content in sauerkraut samples and between *Levilactobacillus* and *Secundilactobacillus* and cadaverine and histamine content in fermented cucumbers.

## Graphical abstract

*Secundilactobacillus* and *Levilactobacillus* were found to be significantly correlated with the content of cadaverine and histamine in the sauerkraut and fermented cucumbers. The high level of biogenic amines in the tested market samples indicates the need to monitor them as an indicator of the hygienic condition of fermented products.

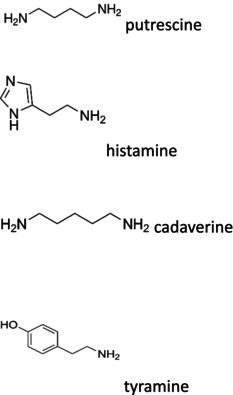

## 1 Introduction

The worldwide popularity of fermented vegetables arises from the common opinion that they are a healthy component of the diet due to the presence of valuable nutrients and potentially probiotic bacteria (Castellone et al. 2021; Diez‐Ozaeta and Astiazaran 2022; Knez et al. 2023). In addition, they are classified as functional foods because they have an additional impact beyond their nutritional function, providing physiological benefits and reducing the risk of lifestyle diseases. Fermented vegetables are obtained during lactic acid bacteria (LAB) fermentation, which is one of the natural methods of food preservation, giving a low‐processed product with unique sensory properties and improved digestibility. In Poland, the most popular fermented products of plant origin are sauerkraut and fermented cucumber, produced both traditionally and industrially. In the case of sauerkraut, white cabbage ( *Brassica oleracea* L.var.*capitata* L.f.*alba*) belonging to the *Brassicaceae* family is most often fermented. Cabbage is a source of vitamins, especially C, B6, B12, potassium, calcium, zinc, and iron. It also contains organic acids, glucosinolates, and dietary fibre. After fermentation, cabbage retains its nutritional value and gains unique taste characteristics. In the case of cucumbers, field cucumbers of the varieties Borus, Julian, Andrus, Octopus, and Śremski are the most often used. Like sauerkraut, they are a source of B vitamins, potassium, iron, calcium, and magnesium. Given the prevalence of both abovementioned fermented vegetables in the diet, their quality is of great importance. The quality of fermentation largely depends on the activity of the microorganisms involved in the process and the raw materials used (Thierry et al. 2024). The research conducted so far on understanding the microbiota of fermented products and their mutual succession has allowed the identification of a large diversity of species involved in lactic acid fermentation. Amongst LAB, representatives of *Lactobacillaceae*, *Streptoccaceae, Enterococcaceae*, *Carnobacteriaceae*, and *Aeroccaceae* were the most frequently mentioned (Xu et al. 2024; Sarengaowa et al. 2024; Junker et al. 2024; Zhang, Zhang, et al. 2023). Moreover, the scientific data showed that to achieve a quality‐stable product with typical taste and aroma, the right sequence of lactic acid bacteria species during fermentation is necessary, e.g., *Leuconostoc mesenteroides*, *Pediococcus pentosaceus* , and *Levilactobacillus brevis* in the initial stage, and subsequently *Lactiplantibacillus plantarum*.

A thorough understanding of the microbial community of fermented vegetables becomes more challenging given the presence of biogenic amines (BAs) in fermented foods (Alvarez and Moreno‐Arribas 2014; Turna et al. 2024). Growing consumer awareness of food safety has led to the need to analyze products for the content of BAs, as these substances may cause adverse health effects, especially in individuals with sensitive immune systems (Wójcik et al. 2021; Omer et al. 2021). Contrary to fresh raw materials, which contain low levels of biogenic amines, consuming fermented foods can risk ingesting excessive amounts of biogenic amines. The formation of BAs occurs in the fermentation process as a result of the metabolic activity of microorganisms, leading to the decarboxylation of amino acids present in the raw material. BAs can be produced by microorganisms in fermented foods such as fermented soy products, fish, cheeses, sausages, and vegetables. Amongst them, soy products, dairy products, fish, and sausages have been the subjects of numerous studies on their BA contents (Turna et al. 2024; Linares et al. 2011; Visciano et al. 2020; Schirone et al. 2022). Relatively less attention has been paid to the problem of biogenic amines in fermented vegetables, although the number of reports is growing (Świder et al. 2020; Alan et al. 2018; Behera et al. 2021; Plengvidhya et al. 2007; Tlais et al. 2022; Gaudioso et al. 2022; Cardinali et al. 2024). The available reports showed increased BA levels in fermented vegetables. Of note, there are currently no regulations directly addressing toxic levels of BAs in fermented foods of plant origin, which may be due to the high variability of individual susceptibility (Ruiz‐Capillas and Herrero 2019; Del Rio et al. 2024; EFSA 2011; Özogul and Özogul 2019). As for studies on the BAs content in sauerkraut and pickled cucumbers, they have mainly focused on determining the BAs level, its changes during storage and reduction using starter cultures (Halasz et al. 1994; Kalač et al. 1999; Rabie et al. 2011; Peñas et al. 2010; Jastrzębska et al. 2023; Špička et al. 2002). The latest reports reported the effect of the raw material (cabbage variety) and yeast, storage conditions, and additives on the accumulation of BAs (Satora et al. 2021; Kosson and Elkner 2010; Majcherczyk and Surówka 2019). Although the conducted studies contributed to understanding the phenomenon of BAs formation in fermented food and new observations were made, the effect of environmental and technological factors on the amino biogenic activity of microorganisms requires thorough research to effectively control BA content and reduce the risk associated with their presence in food.

Hence, the study aimed to determine the BAs content in sauerkraut and fermented cucumbers available on the Polish market to gain insight into the actual level of BAs and to highlight the importance of preventing the occurrence of BAs in fermented foods. Moreover, we attempt to determine the potential correlation between the content of the most important BAs and the existing bacterial population. In addition, other basic parameters influencing the quality of sauerkraut and fermented cucumbers, such as the amount of LAB, pH, acidity, and the amount of organic acids, were also analysed. To our knowledge, the results obtained in this work will fill the research gap regarding BAs in fermented vegetables, as although there are few studies in this field, the correlation between BAs and the native microflora still needs to be clarified. Moreover, in this work, for the first time, an analysis of the correlation between the content of individual biogenic amines and the microbiota in this type of product was performed. Additionally, alpha and beta diversity analyses were conducted to comprehensively assess the microbial diversity within each sample group (alpha diversity) and to compare the overall community composition between the two groups (beta diversity). This study allowed the analysis of existing correlations between LAB and the studied factors in order to better understand them. Considering the variable susceptibility of individuals to BA toxicity, information on their concentration in fermented foods is of great importance for consumers, the food industry and food safety organisations.

## 2 Materials and Methods

### 2.1 Chemicals/Reagents

The LC–MS grade acetonitrile and LC–MS water were purchased from Witko (Łódź, Poland). Di‐sodium tetraborate (borax) ≥ 99% was supplied by Chempur (Piekary Śląskie, Poland). Ammonium formate ≥ 97% and formic acid assay 98%–100% were purchased from Chem‐Lab (Zedelgem, Belgium). Dansyl chloride 97% was purchased from abcr GmbH (Karlsruhe, Germany). Pure trichloroacetic acid was supplied by POCH (Gliwice, Poland). Certified analytical standard (agmatine ≥ 97%, putrescine, histamine, cadaverine, tryptamine, phenylethylamine, tyramine, spermidine, spermine, 1,7‐diamino heptane assay 98%, and ammonium hydroxide solution ~25%), sulphuric acid, lactic acid, acetic acid, propionic acid, butyric acid, and 1‐propanol of analytical grade were supplied by Sigma‐Aldrich (Darmstadt, Germany).

### 2.2 Sample Collection

Ten samples of ready‐to‐eat fermented cucumbers (FO) and 14 samples of sauerkraut (FK) were purchased from local producers and supermarkets located in different regions of Poland, e.g., Masovian, West Pomeranian, Lublin Voivodeships, in the period from October 2023 to October 2024. Two production batches were collected from each producer; for each batch, 2 sample units of 500 g each were collected. There was no information about the addition of starter cultures on the purchased products. The fermented cucumbers collected were traditionally produced in brine, with different amounts of garlic, horseradish, and dill, depending on the recipe. The producers did not provide any further information on the production process of the samples. Samples were stored in a refrigerator (+4°C) immediately after collection and analysed within the next few days. Before the physicochemical and microbiological analysis, the samples were homogenised.

### 2.3 Physicochemical Characteristics

#### 2.3.1 pH

pH of the homogenised samples was determined directly using a pH meter equipped with an In Lab Expert Plus solid electrode (Metler Toledo).

#### 2.3.2 Total Acidity

The acidity of the homogenizate, previously mixed with deionised water in a ratio of 1:5, was determined by titration with 0.1 N sodium hydroxide (NaOH) in the presence of 1% phenolphthalein indicator solution until the colour change persisted for 15 s. The results were expressed as a lactic acid percentage.

All the samples were analysed in duplicate and expressed as mean ± standard deviation.

### 2.4 Determination of Organic Acids by HPLC

The HPLC method was used to determine the organic acids (lactic, acetic, propionic, and butyric) and 1‐propanol in the studied samples. The fermented sample homogenate was centrifuged for 3 min at 150 rpm. The supernatants were filtered through a 0.45 μm filter (Membrane Solutions, USA) before HPLC analysis. The analysis was performed using a Gilson system (Middleton, USA) equipped with an autosampler (GX‐271), a multi‐solvent pump (322), a column oven, and an RI detector (LDC Analytical, Florida, USA). The organic acids were separated using an Aminex 87HP column (300 mm × 7.8 mm, Bio‐Rad Laboratories, Canada, USA) operated at 50°C. Elution was carried out in isocratic mode with sulphuric acid (pH 3.4) as the mobile phase, at a flow rate of 0.8 mL/min. The quantification was based on a refractive index detector and performed with external standards in duplicate and expressed as mean ± standard deviation.

### 2.5 Determination of Biogenic Amines by UPLC‐MS

Biogenic amines (histamine, tyramine, putrescine, cadaverine, tryptamine, spermine, spermidine, agmatine, and 2‐phenethylamine) were analysed according to the method described by Świder et al. 2020, with some modifications. In brief, to obtain a homogeneous material, the sample was ground in a food processor. An aliquot (2 ± 0.01 g) of the fermented sample was weighed into a 50 mL polypropylene centrifuge tube and spiked with 50 μL of 1,7‐diamino heptane internal standard solution (1 mg·mL^−1^). To perform the extraction, 40 mL of 5% trichloroacetic acid was added, and the sample was thoroughly shaken and centrifuged at 10,000*g* for 10 min. The supernatant was filtered through filter paper. Then, the derivatization step was carried out. One millilitre of distilled water, 1 mL of borax solution (5%), and 100 μL of the sample supernatant were mixed in a 15 mL polypropylene tube. Derivatizing agent—dansyl chloride (2.5 mL, 20 mM) dissolved in acetonitrile was added, and the mixture was shaken up and put in a shaking water bath operated at 30°C for 1 h in the dark. Subsequently, 125 μL of ammonia solution (400 mM) was added, and the tube was left intact for 15 min in a dark place. Finally, the mixture was filtered through a 0.45 μm syringe filter into a chromatographic vial for analysis with UPLC‐MS.

#### 2.5.1 UHPLC–MS/MS (Orbitrap) Analysis

Ultra‐high‐performance liquid chromatography coupled to a high‐resolution mass spectrometer Q Exactive Orbitrap Focus MS (Thermo Fisher Scientific, Waltham, MA, US) was used for the analysis. The separation of biogenic amines was performed on a nonporous 100 × 2.1 mm Cortecs UPLC C18 1.6 μm column (Waters, Milford, US) with a flow rate of 0.3 mL·min^−1^ after injecting aliquots (5 μL) of sample solution. The elution was conducted using a gradient system containing solvent A (water/acetonitrile: 90/10) and solvent B (acetonitrile/water: 90/10), each with 5 mM of ammonium formate and 1% formic acid. Gradient A: B (%) was as follows: 90:10 (0–2 min)—waste, 0:100 (2–22 min), 0:100 (22–25 min), 90:10 (25–26 min), and 90:10 (26–28 min). MS was equipped with a heated electrospray ionisation (HESI) source. The analysis was based on scanning in the positive ionisation mode, with the identification of biogenic amines being optimal under the following conditions: 3 kV, capillary temperature: 256°C, sheath gas flow rate: 48, auxiliary gas flow rate: 11, sweep gas flow rate: 2, probe heater temperature: 413°C. Scan range: 200–1200 m/z (Full MS), 80–1000 m/z (AIF). Linearity (as evaluated with standard solution) expressed as the coefficient of determination (r^2^) was at least 0.99 for each analyte. All the samples were analysed in duplicate and expressed as mean ± standard deviation.

Xcalibure 4.2.47 software was used to acquire and analyze data.

The established during validation limits of detection and limits of quantification (LOD and LOQ, respectively) of the studied BA expressed in mg·L^−1^, were as follows: tryptamine (0.09 and 0.29); putrescine (0.01 and 0.03); spermine (0.02 and 0.08); cadaverine (0.02 and 0.06); histamine (0.03 and 0.09); tyramine (0.02 and 0.07); spermidine (0.02 and 0.08); agmatine (0.04 and 0.14); 2‐phenethylamine (0.10 and 0.35).

### 2.6 Viable Counts

Standard plate counting methods based on 10‐fold serial dilutions were used to estimate the number of viable counts of the following groups of microorganisms: total aerobic bacteria on PCA (Plate Count Agar) medium incubated at 30°C for 72 h; presumptive lactobacilli on the MRS (De Man Rogosa and Sharpe) agar medium incubated at 37°C for 48–72 h; *Enterobacteriaceae* on VRBG (Violet Red Bile Glucose) agar incubated at 37°C for 24 h.

The results of two replicates were expressed as the log of colony‐forming units (cfu) per ml of sample and reported as mean ± standard deviation.

### 2.7 DNA Isolation and 16S rDNA Amplicon Sequencing

Total DNA was isolated using the DNeasy PowerSoil Kit (QIAGEN, Hilden, Germany) according to the manufacturer's protocol. DNA purity was measured by the Nanodrop ND‐1000 Spectrophotometer (ThermoFisher Scientific), and DNA concentration was quantified by a Qubit 4.0 Fluorometer using the Qubit dsDNA BR Assay Kit (Invitrogen). Samples were then processed and sequenced using a commercially available kit with a two‐step PCR inhibitor removal system and proprietary modifications developed by genXone S.A. (Złotniki, Poland), a commercial sequencing provider.

The V3–V8 regions of the 16S rRNA gene were amplified (Q5 HiFi polymerase 2× MasterMix—NEB) using the universal primer pair 337F (5′ CAN CCT ACG GGN GGC NGC) and 1391R (5′ GAC GGG CGG TGW GTN CA) (Huse et al. 2007). The PCR assay consisted of 94°C for 1 min, followed by 34 cycles of 94°C for 20 s, 55°C for 30 s, and 65°C for 2 min. The final extension step was 65°C for 5 min. Amplicons were sequenced using Oxford Nanopore Technologies with the native barcoding 1D protocol, following the manufacturer's standard settings. All library preparation, sequencing, and primary quality control steps were performed by genXone according to their internal validated workflows. The obtained amplicons were sequenced using nanopore technology with the Native Barcoding Kit 96 V14 SQK‐NBD114.96 protocol and flow cell type r10.4.1. Sequencing was performed with default settings, using MinKnow software version 5.8.6 and basecalling using Guppy version 7.2.13 and the dna_r10.4.1_e8.2_400bps_5khz_sup model. The taxonomic classification of reads (900–1300 nucleotides long) was performed using the UBLAST/USEARCH algorithm in version v10.0.240_i86linux32 and the NCBI database (16S ribosomal rRNA sequences from bacteria and archaea NCBI).

The obtained reads were taxonomically classified using the UBLAST algorithm, which compared the sequences to the NCBI database through closed‐reference operational taxonomic unit (OTU) picking.

### 2.8 Statistical Analysis

The results were analysed using Statistica version 8.0 software (Statsoft, USA). The Shapiro–Wilk test verified the compatibility of the variable distribution with the normal distribution, whilst the Levene test and Brown‐Forsythe test tested the hypothesis about the homogeneity of variance. An ANOVA and a post hoc analysis (Tukey test) were performed to compare the mean values of particular parameters. The level of significance was 0.05. To determine the strength and direction of the relationship between the studied variables, the nonparametric Spearman rank correlation coefficient (*ρ*) was used. This method allows assessing the monotonic relationship between variables by analysing the relationship between their ranks, not absolute values. The strength of the correlations was interpreted according to commonly accepted criteria: |*ρ*| < 0.2—negligible or very weak correlation, 0.2 ≤ |*ρ*| < 0.4—weak correlation, 0.4 ≤ |*ρ*| < 0.6—moderate correlation, 0.6 ≤ |*ρ*| < 0.8—strong correlation, and |*ρ*| ≥ 0.8—very strong correlation. Statistical significance was set at *p* < 0.05. All analyses were performed using Statistica version 13.0 TIBCO Software Inc. Beta diversity distances were compared within and between groups using a non‐parametric *t*‐test based on 9999 permutations. Model significance was assessed using a PERMANOVA (Permutational Multivariate Analysis of Variance) test. Variance in community composition was also visualised using Principal Coordinates Analysis (PCoA). All analyses and visualisations were performed in R (R Core Team 2025) using the vegan (Oksanen et al. 2020), ggplot2 (Wickham 2016), reshape2 (Wickham 2007), and ggpubr (Kassambara 2020) packages.

## 3 Results and Discussion

### 3.1 Microbial Community

Microbiological analysis showed that the total number of aerobic bacteria and presumptive LAB in the herein analysed samples of sauerkraut (FK) and fermented cucumbers (FO) was in the range of 10^3^–10^8^ cfu·mL^−1^ and above 10^6^ cfu·mL^−1^, respectively (data not shown). In addition, the tests showed the absence of microorganisms from the *Enterobacteriaceae* family. The genetic identification of the bacterial microbiota showed that the dominant group of bacteria was LAB from the *Lactobacillaceae* family.

**FIGURE 1:**
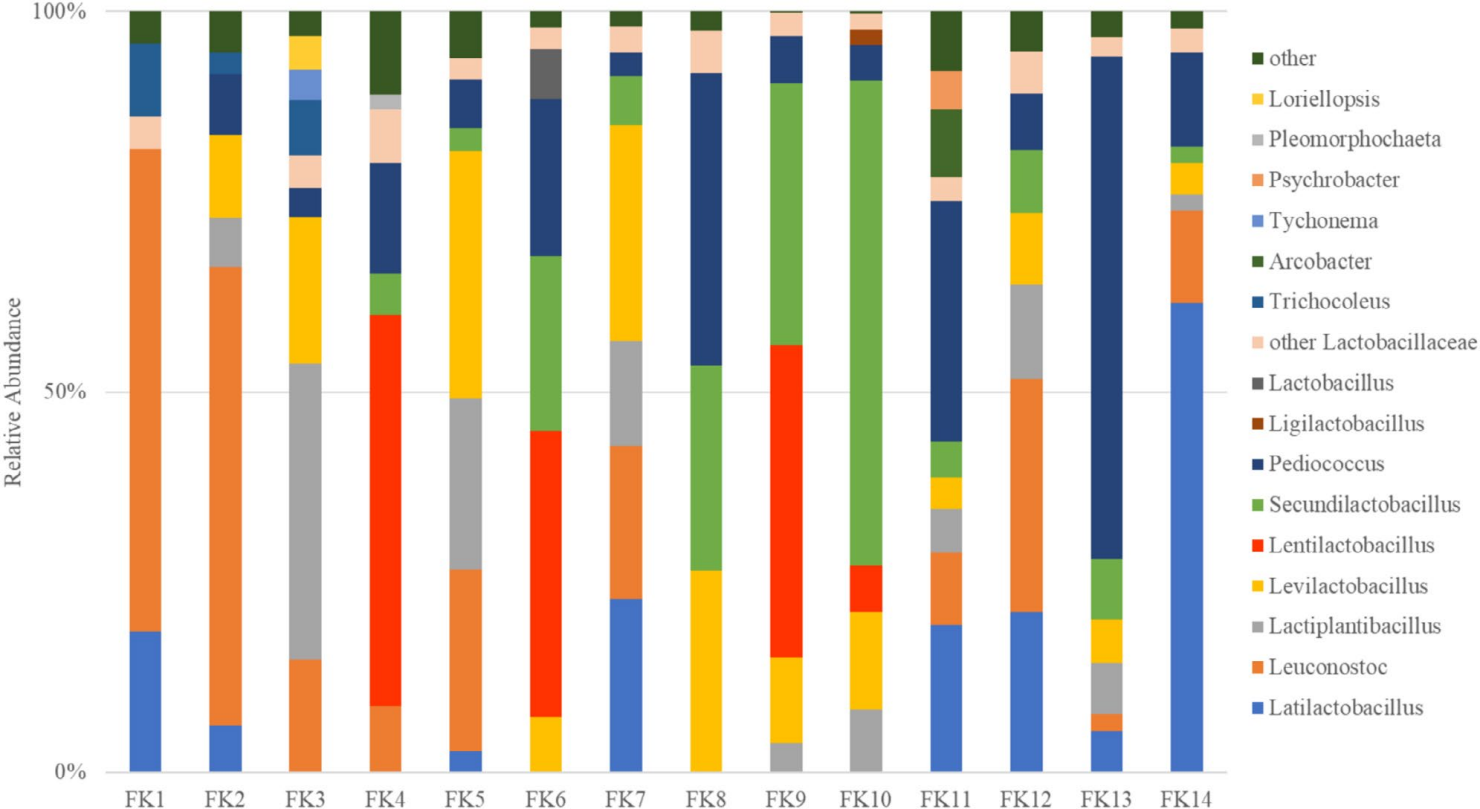
The microbial community of tested sauerkraut samples (FK) at the genus level was revealed by nanopore sequencing. A relative abundance accounted for below 2% has not been shown.

As shown in Figure S1, in sauerkraut, bacteria belonging to this family accounted for more than 90% of the relative abundance of identified bacteria. The remaining identified families, depending on the sample, were *Trichocoleusaceae*, *Microcoleaceae*, *Moraxellaceae, Symphyonemataeae*, *Arcobacteraceae*, and *Spirochaetaceae* constituting less than 2% of the relative abundance. At the genus level (Figure 1), 15 groups of bacteria were identified, of which in most samples the following occurred in significant amounts: *Latilactobacillus*, *Leuconostoc*, *Lactiplantibacillus*, *Levilactobacillus*, *Lentilactobacillus*, *Secundilactobacillus*, *Pediococcus*.

**FIGURE 2:**
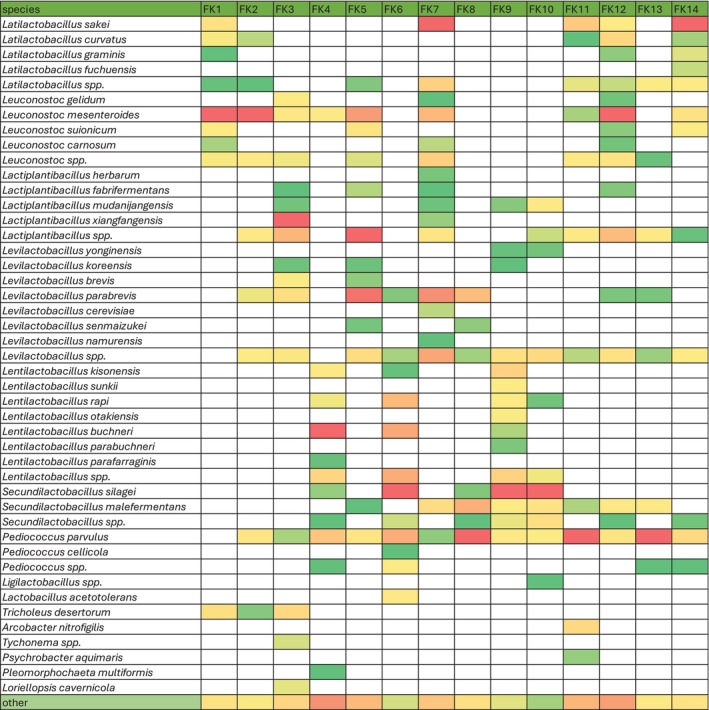
Heatmap representing the microbial community of tested sauerkraut samples (FK) at species level revealed by nanopore sequencing. The lowest relative abundance of species for the given sample is in the darkest green, and the highest relative abundance is in the darkest red.

At the species level, *Pediococcus parvulus* was present in almost all samples (except FK1) in varying amounts (Figure 2). The next species identified in the bacterial microbiota of most samples were *Levilactobacillus* spp., *Lactiplantibacillus* spp., and *Leuconostoc mesenteroides* , in 86%, 64% and 64% of the total number of samples, respectively. Considering the presence of heterofermentative and homofermentative species as well as their varied percentage in the microbial pattern of the tested sauerkraut, it can be assumed that the samples differed in terms of the degree of ensiling (stage of the fermentation process). During the fermentation process, as a result of the metabolic activity of LAB and competition for essential nutrients, pH decreases, inhibiting the growth of pathogenic organisms and others that cause product spoilage. In the first hours of fermentation, the heterofermentative bacteria of the genera *Leuconostoc* and *Lactobacillus* dominate, whilst in the later stages, the homofermentative genus *Pediococcus* and the species *Lactiplantibacillus plantarum* prevail (Plengvidhya et al. 2007). In our study, *Leuc. mesenteroides* dominated the bacterial microbiota of FK1 and FK2, indicating their early fermentation stage, whereas *P. parvulus* dominated in FK8, FK11, and FK13, indicating that these samples were at a more advanced fermentation stage. It is worth noting that the microbial approach did not show the presence of *Lactiplantibacillus plantarum* in any of the sauerkraut samples analyzed; instead, 5 other species amongst the *Lactiplantibacillus* were identified. Moreover, in most of the tested samples, bacterial strains of the species *Secundilactobacillus malefermentans* were identified, and in smaller amounts *Secundilactobacillus silage*, and *Secundilactobacillus* spp., which has recently been described in the literature (Thierry et al. 2024; Plengvidhya et al. 2007; Gaudioso et al. 2022). *S. malefermentans* is a heterolactic LAB species that is tolerant to low temperatures, i.e., 15°C, and ferments mainly glucose. Recently, Tlais et al. (2022) identified *S. malefermentans* during the fermentation process of sauerkraut and found that this species becomes one of the main species of bacterial microbiota in the last weeks of fermentation. Based on their results, the authors suggested that this species, together with *P. parvulus* may play a profound role during the low‐temperature fermentation of sauerkraut.

**FIGURE 3:**
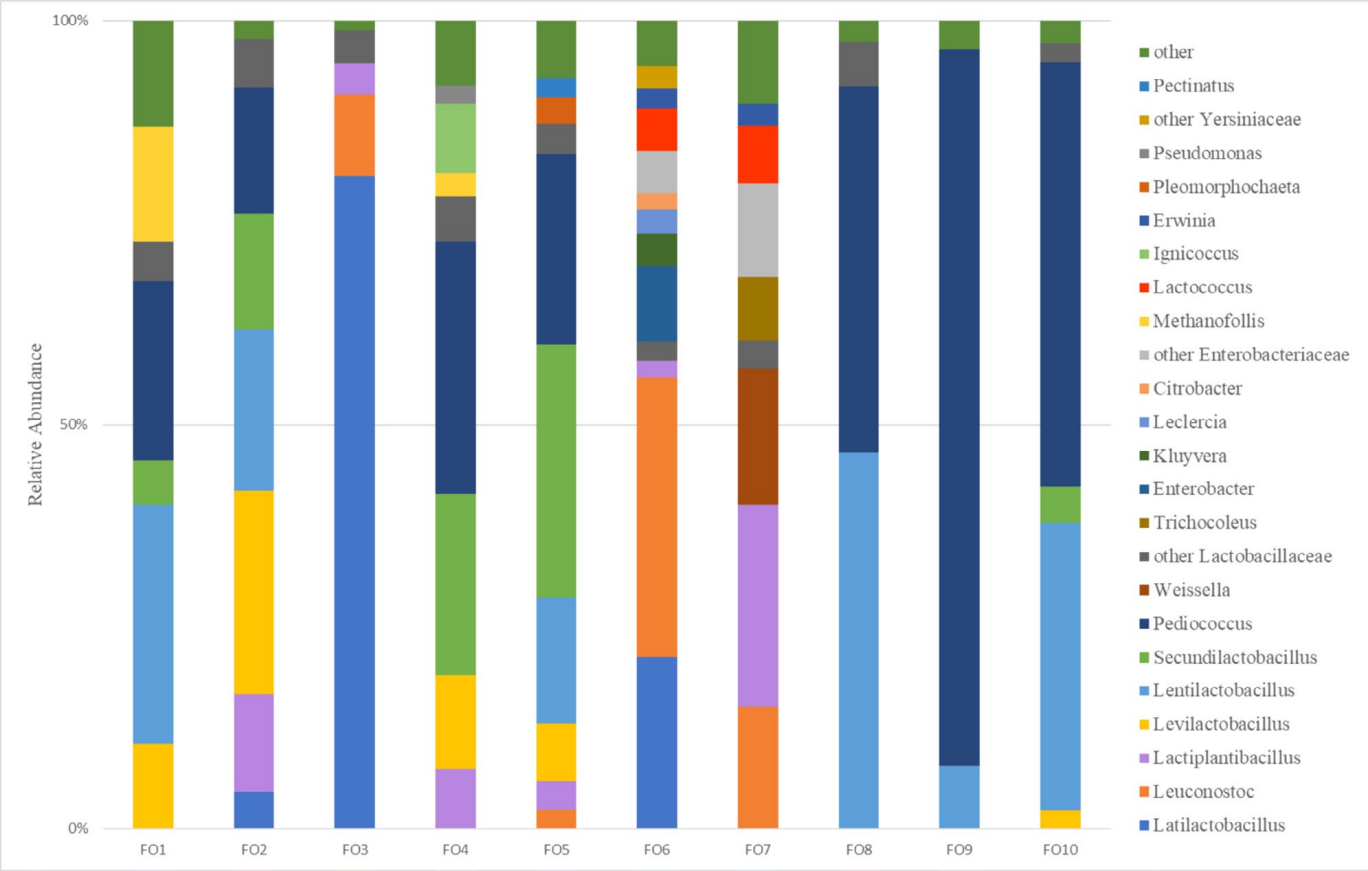
The microbial community of tested fermented cucumber (FO) samples at the genus level was revealed by nanopore sequencing. A relative abundance accounting for below 2% has not been shown.

In the case of fermented cucumbers, bacteria of *Lactobacillaceae* accounted for more than 68% of the relative abundance (Figure S2). In addition to *Lactobacillaceae*, nine other families were identified, e.g., *Trichocoleusaceae, Enterobacteriaceae, Methanomicrobiaceae, Streptococcaceae, Erwiniaceae, Pseudomonadaceae, and Yersiniaceae*, the share of which, however, did not exceed 10% of the relative abundance, except FO6, where the *Enterobacteriaceae* accounted for 23.6%. In the case of *Enterobacteriaceae*, the discrepancy between the results from selective media and the nanopore sequencing data may be due to fundamental methodological differences. Furthermore, the selective composition of VRBG and competition with other microorganisms may have further limited the growth of some *Enterobacteriaceae*. The presence of *Enterobacteriaceae* in a sample indicates reduced microbiological quality. *Enterobacteriaceae* include both opportunistic and potentially pathogenic species, commonly associated with environmental sanitation and hygiene problems (Skowron et al. 2022). At the genus level, 23 groups of bacteria were identified, of which the following were present in significant amounts in most samples: *Latilactobacillus, Leuconostoc, Lactiplantibacillus, Levilactobacillus, Lentilactobacillus, Secundilactobacillus, Pediococcus, Weissella, and Lactococcus* (Figure 3). The content of bacteria of the genus *Erwinia, Enterobacter, Pseudomonas, and Kluyvera* was identified in single cases.

**FIGURE 4:**
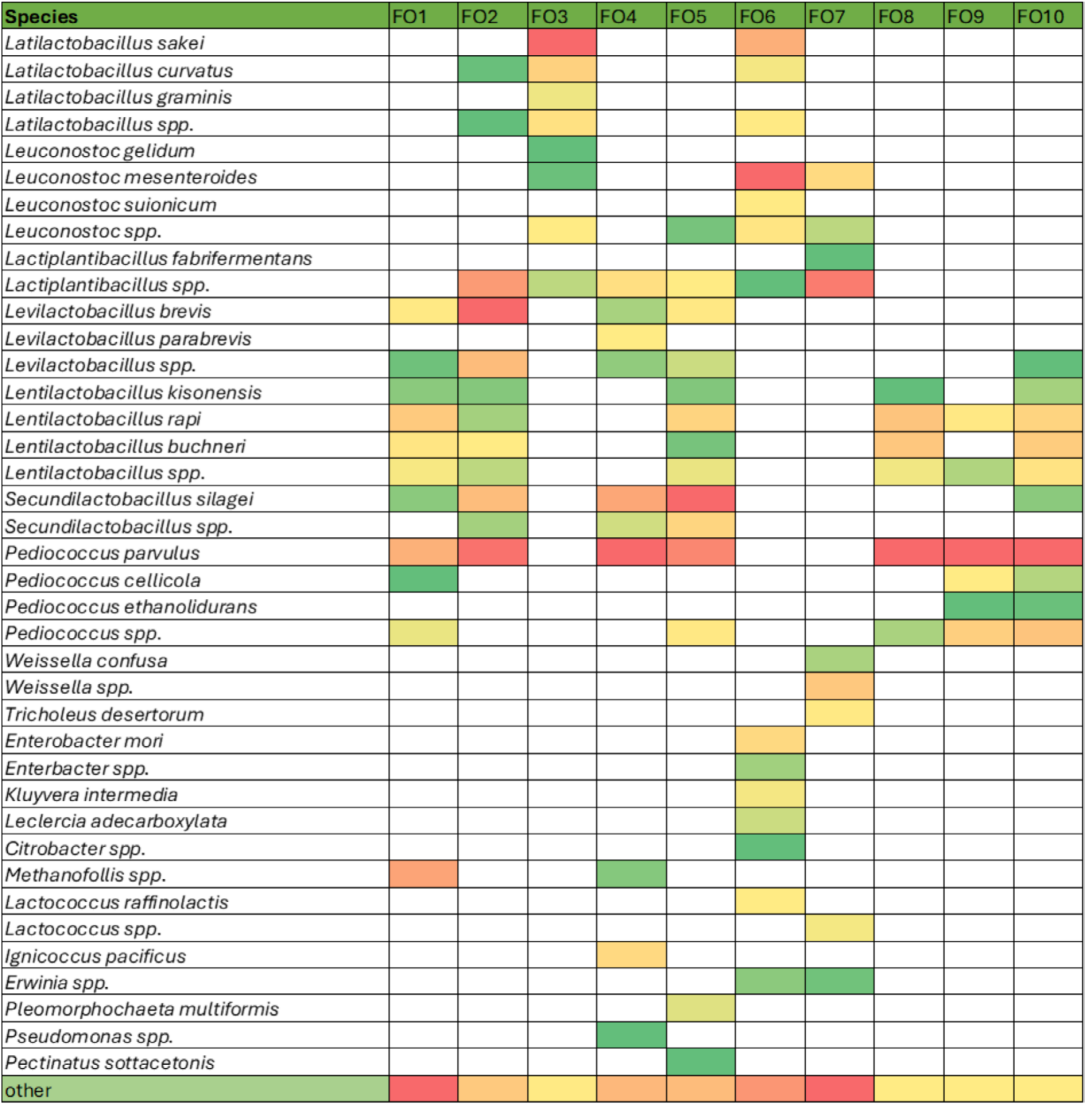
Heatmap of the microbial community of tested fermented cucumber samples (FO) at species level revealed by nanopore sequencing. The lowest relative abundance of species for the given sample is in the darkest green, and the highest relative abundance is in the darkest red.

*Pediococcus* was present in 70% of the total number of samples, and *P. parvulus* was the dominant species amongst this genus (Figure 4). LAB from this species constituted from 13% to 66% of the relative number of identified bacteria, depending on the sample. The next species identified in 60% of the total number of samples were *Lentilactobacillus* spp. and *Lactiplantibacillus* spp. Interestingly, representatives of the genus *Weissella*, often mentioned as members of the microbial community in fermented cucumbers by, for example, Pérez‐Díaz et al. (2020), were identified in only one of our samples. Recently, Cardinali et al. (2024) studied the microbial pattern of fermented cucumbers from selected Polish producers. The authors identified *Lactococcus* and *Streptococcus* as the dominant species of cucumber, whereas the microbial community of brine was dominated by *Lactiplantibacillus*, *Leuconostoc*, *Pediococcus*, *Secundilactobacillus*, and *Lentilactobacillus*. The results of genetic identification obtained in this work are partly similar to these results. The observed differences in the dominance of individual species may be caused by various factors, such as the native microbiota of raw material (cucumber), cucumber varieties, their freshness, fertilisation, harvest period, and duration of the fermentation process. In the case of commercial samples, randomly obtained on the market, the exact procedure of their manufacture is not known. Moreover, they are often obtained in the spontaneous fermentation process driven by local microflora, which is not a controlled process, and thus, it is difficult to standardise.

It is also worth noting that the identification of the microbial community is highly dependent on the sequencing technique used, its accuracy, and its robustness. Genetic identification in our work was performed based on nanopore sequencing, classified as a third‐generation technique. The nanopore technique, in contrast to the previous generation techniques, allows for a significant extension of sequential reads and direct sequencing of native DNA and RNA molecules whilst maintaining high accuracy, efficiency, and short sequencing time (Jain et al. 2018). The accuracy of DNA sequence reads, despite initial shortcomings, is now 95%, thanks to systematic improvements to both the sequencer and the data analysis programmes. The use of V14 reagents and flow cell r10.4.1 in this work ensures high accuracy of the obtained data. The advantages of using nanopore sequencing in 16S rRNA sequencing analyses are confirmed by studies by various research groups. Zhang, Li, et al. (2023) analysed the microbiome of environmental samples by 16S rRNA amplicon sequencing using the Illumina Novaseq PE250, Pacbio Sequel II, and Nanopore PromethION platforms (R9.4.1 and R10.4.1) and showed that the ONT R10.4.1 full‐length 16S flow cell enabled species‐level taxonomic identification of environmental samples with high accuracy. Dommann et al. (2024) compared the nanopore technique for identifying bacterial isolates and profiling microbial communities at the species level with matrix‐based identification using laser desorption ionisation time‐of‐flight mass spectrometry (MALDI‐TOF MS) and Illumina shotgun sequencing. The results showed significant but small differences at the community level between the compared sequencing techniques, and the nanopore technique was identified as a promising alternative to MALDI‐TOF MS and Illumina sequencing.

To assess microbial diversity in sauerkraut and fermented cucumber samples, we calculated alpha and beta diversity indices. These were chosen to provide a comprehensive assessment of diversity patterns across different types of fermented vegetables. Alpha diversity was assessed using the Shannon diversity index, which takes into account both species richness and evenness, providing a robust measure of microbial complexity within a sample (Cassol et al. 2025). For beta diversity, the Bray–Curtis dissimilarity index was used to assess differences in microbial community composition between samples. The Bray–Curtis index is based on relative abundance data and reflects whether there are significant differences in the microbiota between samples (Li and Yue 2016).

**FIGURE 5:**
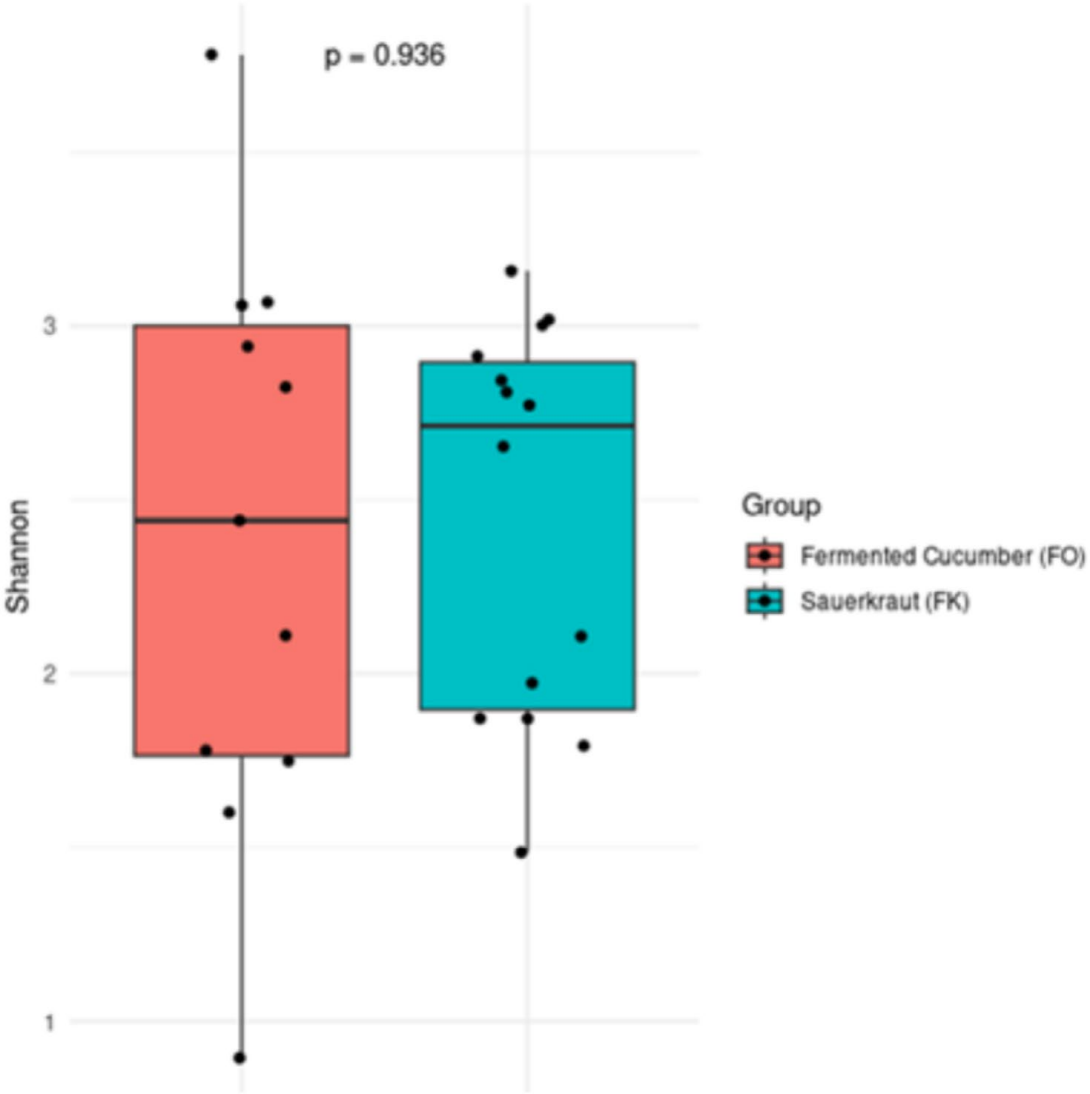
Box plots of alpha‐diversity metrics significantly different (Shannon test; *p*‐value < 0.05) between studied fermented cucumber and sauerkraut samples; *p*‐values are reported in the graph.

The Shannon index did not show any significant differences between groups (*p* > 0.05), although its calculated value was slightly higher for the sauerkraut samples (2.45 ± 0.565) vs. (2.39 ± 0.840 for the fermented cucumbers), indicating a tendency for greater microbial diversity within the sample in this group (Figure 5).

**FIGURE 6:**
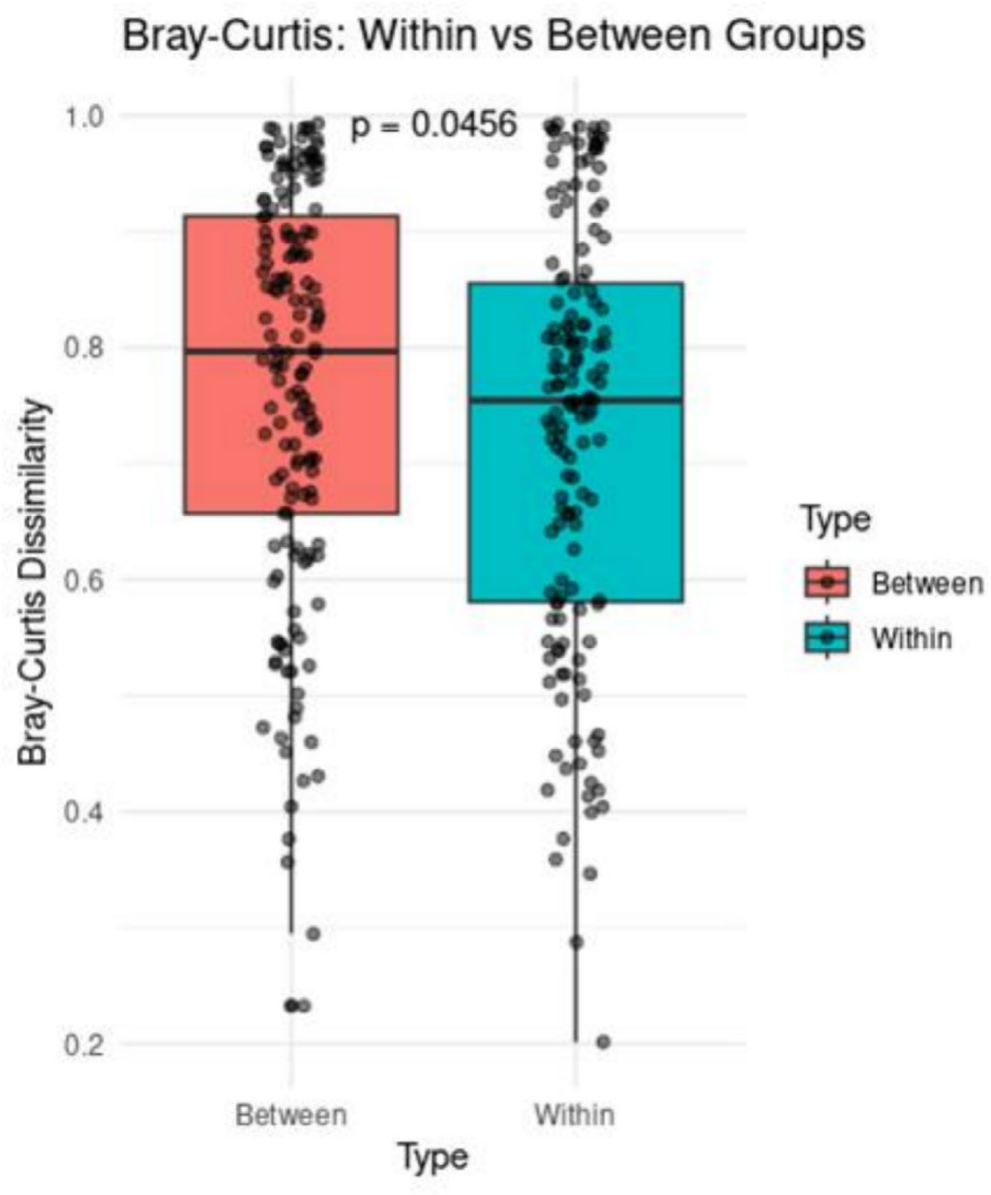
Box plots of beta‐diversity (Bray‐Curtis test; *p*‐value < 0.05) between studied fermented cucumber and sauerkraut samples; *p*‐values are reported in the graph.

The calculated Bray–Curtis dissimilarity index showed that the groups were statistically different (*p* = 0.046) (Figure 6).

The conducted biodiversity analysis using the Shannon and Bray–Curtis indices allowed for a comprehensive comparison of microbial communities between sauerkraut and cucumber samples. The Shannon index did not show statistically significant differences in alpha diversity between groups, although higher mean values in sauerkraut samples suggest a trend towards greater microbial richness and evenness. In contrast, Bray–Curtis dissimilarity analysis identified significant differences in community composition (beta diversity), indicating that the microbial profiles of sauerkraut and cucumber samples are distinct.

### 3.2 Physicochemical Characteristics

The results of physicochemical analyses of randomly selected market samples of sauerkraut (FK) and fermented cucumbers (FO) are presented in Tables S1 and S2, respectively. The pH values differed but were usually within the range desired for good‐quality fermented vegetables included in the Polish standard. The pH of most sauerkraut samples ranged from 3.12 to 3.77. In the case of fermented cucumbers, the pH was between 3.14 and 4.12. The total acidity of half of the total number of sauerkraut samples was at the level required for class I edible foods, i.e., from 1.0% to 1.8% (PN‐A‐75101‐03 1990). In most of the remaining samples, it was above the upper required limit. The total acidity of fermented cucumbers, except for one sample (FO1: 0.4%), was at the required level, i.e., above 0.7%. Some observed deviations (pH and acidity above and below the assumed values for the good quality product) can suggest the loss of control over the process, resulting in, e.g., the activity of undesirable microorganisms. Based on the IQR (interquartile range) analysis, the tested samples of both fermented cucumber and sauerkraut were found to be more diversified in terms of acidity than pH, with the sauerkraut samples having the highest range (Tables S3 and S4). The pH value and acidity of naturally fermented vegetables are shaped during lactic fermentation as a result of the metabolic activity of the native microbiota present, dependent on the content of available carbohydrates (Liu 2003; Paramithiotis et al. 2010). Both parameters are indicators of the quality of the fermentation process. LAB converts carbohydrates into organic acids, mainly lactic acid and acetic acid, which are responsible for lowering pH and increasing acidity (Bangar et al. 2022; Bautista‐Gallego et al. 2020). In addition to the above acids, LAB can produce propionic acid and possibly butyric acid. Regarding the content of organic acids, the analysis of both types of fermented vegetables showed the presence of all the above‐mentioned acids in a wide range of concentrations, which indicates a large diversity of samples available on the market. The dominant acid was lactic acid, followed by acetic acid (Tables S1 and S2). The observed variability of organic acid content between samples results from the previously described species diversity of the samples.

### 3.3 Biogenic Amines

The analysis of biogenic amines (BA) showed that putrescine was the dominant compound in both types of tested samples (Tables 1 and 2, respectively), followed by tyramine, cadaverine, and histamine. For sauerkraut, the content of putrescine was in the range from 4.05 to 339.45 mg·kg^−1^, whilst in fermented cucumber it ranged from 48.17 to 221.32 mg·kg^−1^. Literature data covering studies from different countries show great variability in putrescine content in sauerkraut, e.g.,: 57.50–524.63 mg·kg^−1^ (Poland) (Świder et al. 2020); 100–200 mg·kg^−1^ (Hungary) (Halasz et al. 1994); 108.9 mg·kg^−1^ (Germany) (Mayr and Schieberle 2012); and 98.3–159.6 mg·kg^−1^ (the northeastern region of China) (Liu et al. 2017). In the case of fermented cucumber, Świder et al. (2020) reported putrescine levels in the range of 103.13–286.88 mg·kg^−1^. Putrescine, tyramine, histamine, and cadaverine are the most common BAs found in fermented products (Linares et al. 2011; Schirone et al. 2022). Of these BAs, tyramine and histamine are considered the most harmful to health and exhibit synergistic cytotoxic effects. The other two, putrescine and cadaverine, are not directly toxic but can enhance the effects of histamine. The sum of the four aforementioned biogenic amines (biogenic amine index—BAI) in some cases exceeded the estimated upper limit of toxicity by Świder et al. (2020), specifically the upper limit of toxicity, i.e., 300, which was mostly caused by the high amount of putrescine. Although literature data on the BAs content in fermented vegetables is limited some studies report high BAI values in fermented cucumber and sauerkraut (Świder et al. 2020; Mayr and Schieberle 2012). In the case of sauerkraut, the enhanced level of BA is related to the high content of free amino acids in cabbage. Amongst the other BAs analysed in this work, agmatine and 2‐phenylethylamine were found at low levels, and spermine was below the detection limit of the applied method. Overall, fresh vegetables are marked by relatively low concentrations of BAs, and thus, the main source of BAs in fermented products is microbial production. Microorganisms, mainly LAB, convert free amino acids present in the raw material into biogenic amines through decarboxylation (Karovičová and Kohajdová 2005). It should be emphasised that the ability to create BAs shows not only LAB species, e.g., *Lactobacillus*, *Lactococcus*, and *Enterococcus*, but also pathogenic bacteria from the *Enterobacteriaceae* and *Pseudomonasaceae* families (Barbieri et al. 2019). A positive correlation between the number of *Enterobacteriaceae* species tested and the concentration of selected BAs was reported by Marino et al. (2000). The spontaneous fermentation process is, therefore, a natural way to increase the amount of biogenic amines in the traditionally fermented product. The growing concern about the presence of BAs in fermented foods generates interest in studying the correlated microbiota. Several studies have been performed to analyse the in vitro decarboxylase activity of varied LAB species (Paramithiotis et al. 2022; Lee et al. 2021; Sarkadi 2017; Russo et al. 2019; Septembre‐Malaterre et al. 2018). In our work, we performed Spearman correlation analysis between the content of the four prevailing BAs and the identified dominant bacterial genera to get more insight into the role of the microbiological community on the BAs values in sauerkraut and fermented cucumber (Table 3).

**TABLE 1:** Content of individual biogenic amines and the calculated BAI values for the commercial sauerkraut (FK).

Sample	Agmatine [mg·L^−1^]	Putrescine [mg·L^−1^]	Tryptamine [mg·L^−1^]	Histamine [mg·L^−1^]	2‐Phenyl‐ethylamine [mg·L^−1^]	Cadaverine [mg·L^−1^]	Tyramine [mg·L^−1^]	Spermidine [mg·L^−1^]	Spermine [mg·L^−1^]	Total amount [mg·L^−1^]	BAI [mg·L^−1^]
FK1	0.47^b,c,d^ (0.07)	22.76^a,b^ (2.73)	7.81^c,d^ (0.86)	53.83^g^ (4.84)	< 0.35^a^	2.20^a,b^ (0.26)	26.29^d^ (2.89)	5.44^b,c,d,e^ (0.49)	< 0.08	118.80	105
FK2	1.05^f^ (0.16)	257.91^h^ (30.95)	10.51^d,e^ (1.16)	57.81^g,h^ (5.20)	1.78^c^ (0.27)	35.62^e^ (4.27)	40.36^f^ (4.44)	5.34^b,c,d,e^ (0.48)	< 0.08	410.38	392
FK3	0.15^a^ (0.02)	4.05^a^ (0.49)	0.82^a^ (0.09)	< 0.09^a^	< 0.35^a^	1.51^a^ (0.18)	< 0.07^a^	2.87^a^ (0.26)	< 0.08	9.40	6
FK4	0.42^b,c^ (0.06)	54.02^b,c,d^ (6.48)	5.96^b,c^ (0.66)	12.07^c^ (1.09)	1.26^b^ (0.19)	25.47^d^ (3.06)	16.65^c^ (1.83)	2.24^a^ (0.20)	< 0.08	118.07	108
FK5	1.30^f^ (0.20)	137.02^f,g^ (16.44)	2.03^a^ (0.22)	9.34^b,c^ (0.84)	1.17^b^ (0.17)	9.67^c^ (1.16)	35.42^e^ (3.90)	3.86^a,b^ (0.35)	< 0.08	199.81	191
FK6	0.39^a,b,c^ (0.06)	82.08^d,e^ (9.85)	3.85^a,b^ (0.42)	25.61^e^ (2.30)	1.18^b^ (0.18)	4.94^a,b,c^ (0.59)	29.86^d,e^ (3.28)	4.24^a,b,c^ (0.38)	< 0.08	152.15	142
FK7	< 0.14^a^	171.60^g^ (20.59)	0.93^a^ (0.10)	17.74^d^ (1.60)	< 0.35^a^	49.84^f^ (5.98)	6.87^b^ (0.76)	5.52^b,c,d,e^ (0.50)	< 0.08	252.50	246
FK8	0.35^a,b^ (0.05)	271.52^h^ (32.58)	17.91^g,h,i^ (1.97)	96.20^i^ (8.66)	3.46^e^ (0.52)	58.42^g^ (7.01)	84.42^h^ (9.29)	8.80^f^ (0.79)	< 0.08	540.73	511
FK9	< 0.14^a^	259.46^h^ (31.13)	15.48^f,g,h^ (1.70)	43.49^f^ (3.91)	2.82^d^ (0.42)	80.53^h^ (9.66)	59.43^g^ (6.54)	5.06^b,c,d^ (0.46)	< 0.08	466.27	443
FK10	< 0.14^a^	278.22^h^ (33.39)	18.25^h,i^ (2.01)	63.48^h^ (5.71)	2.82^d^ (0.42)	117.65^i^ (14.12)	81.84^h^ (9.00)	6.31^c,d,e^ (0.57)	< 0.08	568.57	541
FK11	0.61^c,d,e^ (0.09)	103.79^e,f^ (12.45)	12.62^e,f^ (1.39)	19.60^d^ (1.76)	1.33^b,c^ (0.20)	7.84^b,c^ (0.94)	33.92^e^ (3.73)	7.14^d,e,f^ (0.64)	< 0.08	186.85	165
FK12	0.73^e^ (0.11)	29.42^a,b,c^ (3.53)	20.14^i^ (2.22)	2.79^a^ (0.25)	2.90^d^ (0.43)	7.62^a,b,c^ (0.91)	15.11^c^ (1.66)	6.78^d,e,f^ (0.61)	< 0.08	85.49	55
FK13	0.71^d,e^ (0.11)	68.48^c,d,e^ (8.22)	15.22^f,g,h^ (1.67)	17.86^d^ (1.61)	< 0.35^a^	10.63^c^ (1.28)	31.47^d,e^ (3.46)	7.35^e,f^ (0.66)	< 0.08	151.72	128
FK14	0.75^e^ (0.11)	339.45^i^ (40.73)	14.63^f,g^ (1.61)	4.88^a,b^ (0.44)	< 0.35^a^	6.89^a,b,c^ (0.83)	60.77^g^ (6.68)	7.29^e,f^ (0.66)	< 0.08	434.66	412

*Note:* BAI, biogenic amine index calculated acc. to Świder et al. (2020); BAI, putrescine + cadaverine + histamine + tyramine. Values are expressed as means ± standard deviations (given in brackets). Different letters indicate significant differences amongst the mean values according to the least significant difference test (*p* ≤ 0.05).

**TABLE 2:** Content of individual biogenic amines and the calculated BAI values for the commercial fermented cucumber (FO).

Sample	Agmatine [mg·L^−1^]	Putrescine [mg·L^−1^]	Tryptamine [mg·L^−1^]	Histamine [mg·L^−1^]	2‐Phenyl‐ethylamine [mg·L^−1^]	Cadaverine [mg·L^−1^]	Tyramine [mg·L^−1^]	Spermidine [mg·L^−1^]	Spermine [mg·L^−1^]	Total amount [mg·L^−1^]	BAI [mg·L^−1^]
FO1	< 0.14^a^	48.17^a^ (5.78)	2.51^a^ (0.28)	11.92^a^ (1.07)	1.45^a,c^ (0.22)	24.05^a^ (2.89)	20.11^c^ (2.21)	3.55^b^ (0.32)	< 0.08	111.76	104
FO2	< 0.14^a^	221.32^b^ (26.56)	15.18^d^ (1.67)	44.80^e^ (4.03)	3.28^b^ (0.49)	74.72^d^ (8.97)	56.39^d^ (6.20)	9.51^a^ (0.86)	< 0.08	425.20	397
FO3	0.20^b^ (0.03)	54.91^a^ (6.59)	3.38^a^ (0.37)	0.87^c^ (0.08)	< 0.35^a^	< 0.06^c^	40.39^a^ (4.44)	11.78^d^ (1.06)	< 0.08	111.53	96
FO4	< 0.14^a^	147.99^d,e^ (17.76)	11.42^c^ (1.26)	33.79^b^ (3.04)	2.94^b^ (0.44)	54.62^b^ (6.55)	43.81^b^ (4.82)	7.55^a^ (0.68)	< 0.08	302.12	280
FO5	< 0.14^a^	114.88^c^ (13.79)	7.74^b^ (0.85)	34.80^b^ (3.13)	7.14^d^ (1.07)	22.71^a^ (2.73)	43.68^a,b^ (4.80)	7.81^a^ (0.70)	< 0.08	238.76	216
FO6	< 0.14^a^	176.49^b,e^ (21.18)	1.23^a^ (0.14)	17.89^a,d^ (1.61)	< 0.35^a^	51.15^b^ (6.14)	7.90^e^ (0.87)	4.07^b^ (0.37)	< 0.08	258.73	253
FO7	1.45^c^ (0.15)	50.39^a^ (6.05)	0.77^a^ (0.08)	1.19^c^ (0.11)	1.63^a,c^ (0.24)	20.09^a^ (2.41)	38.32^a^ (4.22)	9.12^a,d^ (0.82)	< 0.08	121.51	110
FO8	< 0.14^a^	80.36^a^ (9.64)	7.97^b^ (0.88)	11.59^a^ (1.04)	< 0.35^a^	22.69^a^ (2.72)	21.45^c^ (2.36)	4.99^b,c^ (0.45)	< 0.08	149.05	136
FO9	0.16^b^ (0.03)	187.61^b^ (22.51)	3.52^a^ (0.39)	30.5^b^ (2.74)	3.28^b^ (0.49)	17.98^a^ (2.16)	54.75^d^ (6.02)	8.30^a^ (0.75)	< 0.08	306.10	291
FO10	0.19^b^ (0.03)	132.57^c,d^ (15.91)	3.69^a^ (0.41)	22.36^d^ (2.01)	2.30^b,c^ (0.34)	17.47^a^ (2.10)	46.21^b^ (5.08)	7.18^a,c^ (0.65)	< 0.08	231.97	219

*Note:* BAI, biogenic amine index calculated acc. to Świder et al. (2020), BAI, putrescine + cadaverine + histamine + tyramine. Values are expressed as means ± standard deviations (given in brackets). Different letters indicate significant differences amongst the mean values according to the least significant difference test (*p* ≤ 0.05).

**TABLE 3:** Spearman's moment rank correlation (*R*), *p* < 0.05 (italics), between the dominant genera and content of the main biogenic amines in the studied (a) sauerkraut (FK) and (b) fermented cucumbers (FO).

(a)							
Biogenic amine	LAB genera						
	*Latilacto‐bacillus*	*Leuconostoc*	*Lactiplanti‐bacillus*	*Levilacto‐bacillus*	*Lentilacto‐bacillus*	*Secundilacto‐bacillus*	*Pediococcus*
Putrescine	0.07 (*0.822*)	−0.360 (*0.206*)	−0.118 (*0.688*)	0.319 (*0.266*)	0.052 (*0.859*)	0.333 (*0.244*)	0.160 (*0.584*)
Cadaverine	−0.263 (*0.363*)	−0.447 (*0.109*)	0.007 (*0.982*)	0.392 (*0.166*)	0.333 (0.244)	0.620 (*0.018*)	0.072 (*0.805*)
Histamine	−0.304 (*0.290*)	−0.335 (*0.241*)	−0.475 (*0.086*)	0.055 (*0.852*)	0.206 (*0.479*)	0.373 (*0.189*)	0.064 (*0.829*)
Tyramine	−0.169 (*0.563*)	−0.471 (*0.089*)	−0.304 (*0.290*)	0.130 (*0.658*)	0.088 (*0.764*)	0.417 (0.138)	0.332 (*0.246*)

(b)							
Biogenic amine	LAB genera						
	*Latilacto‐bacillus*	*Leuconostoc*	*Lactiplanti‐bacillus*	*Levilacto‐bacillus*	*Lentilacto‐bacillus*	*Secundilacto‐bacillus*	*Pediococcus*
Putrescine	0.231 (*0.520*)	−0.218 (*0.544*)	0.056 (*0.877*)	0.226 (*0.529*)	−0.069 (*0.850*)	0.149 (*0.682*)	0.288 (*0.419*)
Cadaverine	0.052 (*0.886*)	−0.143 (*0.693*)	0.313 (*0.379*)	0.666 (*0.035*)	−0.031 (*0.932*)	0.549 (*0.09*)	0.549 (*0.623*)
Histamine	−0.171 (*0.636*)	−0.416 (*0.231*)	0.094 (*0.797*)	0.705 (*0.023*)	0.156 (*0.666*)	0.743 (*0.014*)	0.386 (*0.270*)
Tyramine	−0.169 (*0.636*)	−0.519 (*0.124*)	0.100 (*0.783*)	0.323 (*0.352*)	0.09 (*0.810*)	0.304 (*0.393*)	0.546 (*0.102*)

*Note:* Red color indicates statistically significant values.

In general, the obtained results showed a mostly positive value of the correlation coefficient between the BA content and the tested LAB genera, e.g., *Lactiplantibacillus, Levilactobacillus*, *Lentilactobacillus*, *Secundilactobacillus*, and *Pediococcus*. However, a significant positive correlation was found only between cadaverine content and *Secundilactobacillus* and *Levilactobacillus* for sauerkraut and fermented cucumbers, respectively. Furthermore, for fermented cucumbers, other significant positive correlations were found between histamine content and *Levilactobacillus* and *Secundilactobacillus*. A negative correlation coefficient was noted for *Leuconostoc* and all four tested amines in both the sauerkraut and fermented cucumber samples, and *Latilactobacillus* and cadaverine, histamine, and tyramine in the sauerkraut samples and histamine and tyramine in the fermented cucumber samples. Of note, in the Spearman correlation analysis, the most important aspect is the significance of the correlation; the sign of the coefficient only informs about the direction of the correlation. In addition, the size of the coefficient is influenced by extreme values and outliers.

As for cadaverine, Lorencová et al. (2012), based on in vitro studies of decarboxylase activity of 25 LAB from dairy products and seven strains of *Lev. brevis* from beer, showed that these strains produced small amounts of cadaverine (< 50 mg L^−1^). These results were in contrast to the suggestions of other authors who stated that LAB do not secrete any lysine decarboxylase (EC 4.1.1.18) catalysing the conversion of lysine to cadaverine. Although the study of Lorencová was performed in the MRS medium our results are in line with them, and the discrepancies with other authors may probably result, as it has been suggested, from the existence of genes for arginine, lysine, and ornithine decarboxylase (EC: 4.1.1.x), which may be encoded in the genomes of some LAB. These results, however, clearly indicate the varied ability of LAB strains for the synthesis of the above‐mentioned enzymes and the resulting different amino acids' metabolic pathways. The choice of decarboxylation route depends largely on environmental conditions such as temperature and pH, as well as on the salt content, additives, and storage conditions (Świder et al. 2020). It has been found that low pH promotes decarboxylase activity, which is a defence mechanism of microorganisms against adverse environmental conditions (Comas‐Basté et al. 2019; Moreno‐Arribas and Lonvaud‐Funel 2001). Romano et al. (2014) showed that *Lev. brevis* strains isolated from different environments produce putrescine via ornithine decarboxylation to maintain intracellular pH under acid‐stress conditions.

Regarding the sauerkraut samples studied in our study, they were characterised by high variability in terms of pH, acidity, organic acid content, and BAs level. The highest putrescine content was found for samples with pH 3.75 (FK14), 3.72 (FK10), and 3.67 (FK2), as well as for two samples with lower pH, i.e., 3.12 (FK8) and 3.48 (FK9) (Tables S1 and 1). FK8 was characterised by the highest acidity (4.6%), lactic acid content, and high acetic acid content amongst the tested samples. Moreover, the highest histamine and tyramine contents were also determined in this sample. Considering the relative abundance of bacterial microbiota, heterofermentative bacteria of the genus *Leuconostoc* (63.4%) dominated in this sample, followed by *Levilactobacillus* (26.6%) and *Secundilactobacillus* (26.9%) (Figure 1). Interestingly, *Leuconostoc* also dominated in sample FK13 (60.2%), but the remaining genera mentioned above were at a much lower level, and the BAs levels determined for this sample were lower than FK8. However, FK13 had a similarly high content of lactic and acetic acids. The highest cadaverine content was found for FK10 (pH of 3.72). Overall, this sample was characterised by the highest total BAs content amongst the analysed sauerkraut samples and the highest acetic acid content. The bacterial community of this sample was dominated by S*ecundilactobacillus* (63.8%). High acetic acid content was measured for FK14, which, as previously described, was characterised by the highest putrescine content. Of the remaining BAs, this sample had a relatively high tyramine content, whilst cadaverine and histamine were at low levels. The dominant genera in this sample were *Leuconostoc* (30.6%) and *Latilactobacillus* (21.1%).

In the case of fermented cucumbers, the highest level of putrescine, cadaverine, histamine, and tyramine was found for FO2 (Table 2), which had a pH of 3.52 and the highest content of lactic and acetic acid (Table S2). The highest level of tyramine and a relatively high level of putrescine were detected in the case of FO9 with the highest acidity, similar to the case of the sauerkraut sample FK8. Relatively high levels of putrescine and cadaverine were obtained for FO6, with the highest pH of 4.12 amongst the tested samples. In this sample, the presence of *Enterobacteriaceae* bacteria was identified at the highest level amongst the analysed samples (23.6%) (Table S2). Gram‐negative *Enterobacteriaceae* are one of the major bacterial families, along with *Pseudomonadaceae* and *Shewanellaceae*, associated with the production of putrescine and cadaverine (EFSA 2011).

The highest BAs concentrations were recorded for samples characterised by high acidity and the highest content of acetic and lactic acid. This follows the suggested mechanism of BAs production through the decarboxylation of amino acids, enabling the survival of acid stress. However, some deviations were observed, which are probably the result of the complexity of the native microbial community and the strain‐dependent ability to activate decarboxylase under given environmental conditions. As can be seen in the example of the two types of fermented vegetables analysed in this work, the type of raw material subjected to fermentation and its specific composition (e.g., content of sugars, amino acids) also significantly determine the differences in the content of produced BAs. Thus, the analysis of spontaneously fermented foods allows for a broader perspective on the complexity of the discussed phenomenon and adds value to the general understanding of the problem of BAs reduction in food. Another important result of this work is the finding of a significant positive correlation between the content of cadaverine and histamine and bacteria from the previously less common genus, *Secundilactobacillus*. Our findings and studies by Tlais et al. (2022) and Zhang, Zhang, et al. (2023) suggest that this genus is becoming an increasingly important representative of LAB in the fermentation process.

## 4 Conclusions

The study showed that samples of sauerkraut and cucumbers available on the Polish retail market are characterised by different physicochemical compositions and content of biogenic amines, which may be caused by differences in the composition of microbiological communities (beta diversity). The results of the analyses allowed the assessment of the quality status of these fermented products, especially in terms of BAs. Moreover, for the first time, we demonstrated the existence of significant correlations between the presence of the species *Secundilactobacillus* and *Levilactobacillus* and the content of cadaverine and histamine. Although the issue of the presence of BAs in fermented food is relatively well known, it is difficult to prevent their accumulation, considering the specificity of the fermentation process and the microorganisms involved in it. Therefore, it is necessary to deepen our understanding of the amino acid (BA) production capacity of the microbial community involved in fermentation, and monitoring the amino acid (BA) content in fermented vegetables is strongly recommended. Finally, the limitations of the studies conducted must be taken into account, as establishing a clear correlation between pH, acidity, specific types of lactic acid bacteria and the content of specific amines is difficult due to the complexity of the matrix of fermented foods.

## Author Contributions

**Renata Choińska:** investigation, methodology, data curation, validation, visualization, writing – original draft. **Katarzyna Piasecka‐Jóźwiak:** resources, supervision, writing – review and editing. **Olga Świder:** investigation, writing – review and editing. **Agata Żak‐Kułakowicz:** investigation. **Karol Włodarczyk** and **Juliusz Załuski:** investigation. All authors read and approved the final manuscript.

## Funding

This work was supported by the Ministry of Agriculture, DRE.prz.070.2.2024.

## Conflicts of Interest

The authors declare no conflicts of interest.

## Supporting information


**Figure S1:** The microbial community of tested sauerkraut samples (FK) at the family level revealed by nanopore sequencing. A relative abundance accounted for below 2% has not been shown.
**Figure S2:** The microbial community of tested fermented cucumber (FO) samples at the family level revealed by nanopore sequencing. A relative abundance accounted for below 2% has not been shown.

**Table S1:** Physicochemical characteristics and the content of selected organic acids of the studied sauerkraut (FK) samples.
**Table S2:** Physicochemical characteristics and the content of selected organic acids of the studied fermented cucumber (FO) samples.
**Table S3:** IQR analysis for pH and acidity of sauerkraut (FK) and fermented cucumber (FO) samples.
**Table S4:** IQR analysis for content of organic acid of sauerkraut (FK) and fermented cucumber (FO) samples.

## Data Availability

The datasets generated and/or analysed during the current study are available in the NCBI BioSample repository, SAMN47466429; SAMN47466430; SAMN47466431; SAMN47466432; SAMN47466433; SAMN47466434; SAMN47466435; SAMN47466436; SAMN47466437; SAMN47466438; SAMN47466439; SAMN47466440; SAMN47466441; SAMN47466442; https://www.ncbi.nlm.nih.gov/sra/PRJNA1238322, and SAMN47467875; SAMN47467876; SAMN47467877; SAMN47467878; SAMN47467879; SAMN47467880; SAMN47467881; SAMN47467882; SAMN47467883; SAMN47467884; https://www.ncbi.nlm.nih.gov/sra/PRJNA1238285.
