# Supplementary material for: Microbiota and Quality Indexes of Commercial Sauerkraut and Fermented Cucumbers
Source: Environ Microbiol Rep. 2025 Dec 12;17(6):e70250. doi: 10.1111/1758-2229.70250 (PMC12699377; doi:10.1111/1758-2229.70250)
Supplement: Supplementary file 1 — Figure S1: The microbial community of tested sauerkraut samples (FK) at the family level revealed by nanopore sequencing. A relative abundance accounted for below 2% has not been shown. Figure S2: The microbial community of tested fermented cucumber (FO) samples at the family level revealed by nanopore sequencing. A relative abundance accounted for below 2% has not been shown. [file EMI4-17-e70250-s001.docx]

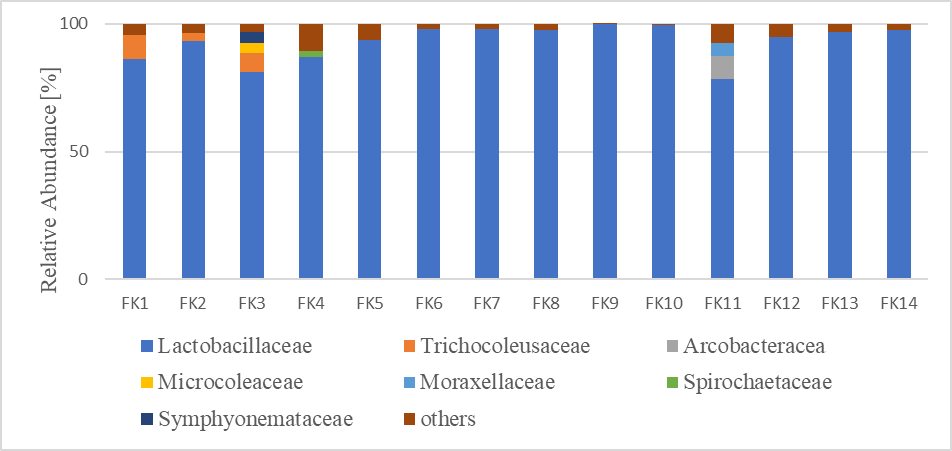


Figure S1. The microbial community of tested sauerkraut samples (FK) at the family level revealed by nanopore sequencing. A relative abundance accounted for below 2 % has not been shown.


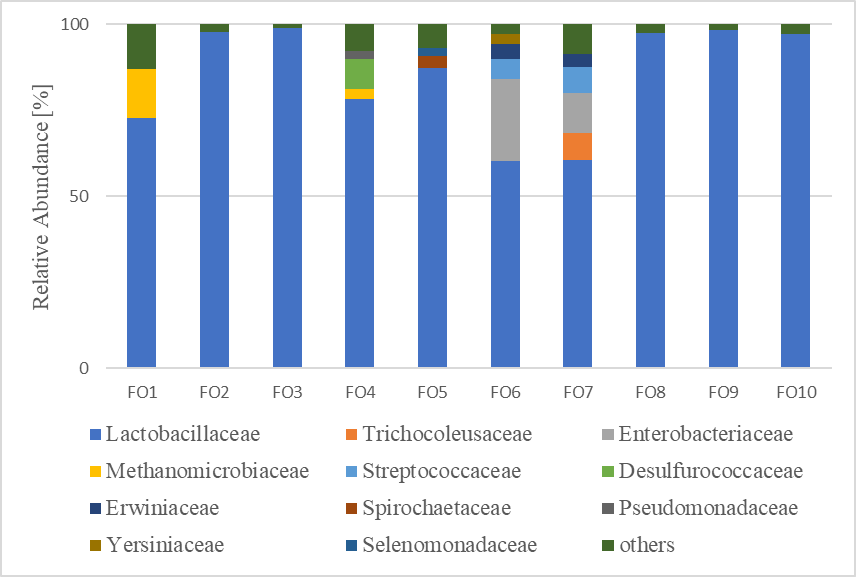


Figure S2. The microbial community of tested fermented cucumber (FO) samples at the family level revealed by nanopore sequencing. A relative abundance accounted for below 2 % has not been shown.
