# Supplementary material for: Microbiota and Quality Indexes of Commercial Sauerkraut and Fermented Cucumbers
Source: Environ Microbiol Rep. 2025 Dec 12;17(6):e70250. doi: 10.1111/1758-2229.70250 (PMC12699377; doi:10.1111/1758-2229.70250)
Supplement: Supplementary file 2 — Table S1: Physicochemical characteristics and the content of selected organic acids of the studied sauerkraut (FK) samples. Table S2: Physicochemical characteristics and the content of selected organic acids of the studied fermented cucumber (FO) samples. Table S3: IQR analysis for pH and acidity of sauerkraut (FK) and fermented cucumber (FO) samples. Table S4: IQR analysis for content of organic acid of sauerkraut (FK) and fermented cucumber (FO) samples. [file EMI4-17-e70250-s002.docx]

Table S1. Physicochemical characteristics and the content of selected organic acids of the studied sauerkraut (FK) samples.

|  | **FK1** | **FK2** | **FK3** | **FK4** | **FK5** | **FK6** | **FK7** | **FK8** | **FK9** | **FK10** | **FK11** | **FK12** | **FK13** | **FK14** |
| --- | --- | --- | --- | --- | --- | --- | --- | --- | --- | --- | --- | --- | --- | --- |
| **pH** | **3.77** ±0.01 | **3.67** ±0.01 | **3.58** ±0.01 | **3.55** ±0.01 | **3.48** ±0.01 | **3.52** ±0.01 | **3.67** ±0.01 | **3.12** ±0.01 | **3.48** ±0.01 | **3.72** ±0.01 | **3.53** ±0.01 | **3.76** ±0.01 | **3.38** ±0.01 | **3.75** ±0.01 |
| **acidity** [%] | **3.60** ±0.01 | **2.59** ±0.01 | **1.86** ±0.01 | **2.19** ±0.01 | **1.31** ±0.01 | **1.33** ±0.01 | **4.49** ±0.01 | **4.60** ±0.01 | **1.65** ±0.01 | **1.75** ±0.01 | **1.74** ±0.01 | **1.58** ±0.01 | **2.68** ±0.01 | **1.81** ±0.01 |
| **lactic acid** [mg·L^-1^] | **4635** ±174 | **4028** ±140 | **2330** ±144 | **2432** ±121 | **5279** ±171 | **1792** ±122 | **9686** ±314 | **16046** ±212 | **6035** ±144 | **5964** ±149 | **8899** ±128 | **7605** ±302 | **15334** ±455 | **9697** ±166 |
| **acetic acid** [mg·L^-1^] | **1751**  ±135 | **1430** ±32 | **631** ±32 | **1200** ±62 | **1569** ±23 | **1424** ±34 | **2590** ±51 | **2961** ±15 | **818** ±29 | **3012** ±34 | **1883** ±11 | **2865** ±114 | **2190** ±53 | **2635** ±29 |
| **propionic acid** [mg·L^-1^**]** | **173** ±14 | <49 | <49 | **178** ±11 | **245** ±12 | **227** ±17 | **296** ±15 | **1454** ±17 | **341** ±26 | **671** ±10 | **389** ±20 | **269** ±13 | **409** ±13 | **366** ±10 |
| **butyric acid** [mg·L^-1^] | **5956** ±154 | **11865** ±189 | **1708** ±28 | **329** ±10 | **1830** ±15 | **2228**  ±33 | **2991** ±94 | **3071**  ±97 | **1677** ±50 | **4713**  ±16 | **3897**  ±35 | **520**  ±10 | **573** ±19 | **715**  ±14 |

Values are expressed as means ± standard deviations

Table S2. Physicochemical characteristics and the content of selected organic acids of the studied fermented cucumber (FO) samples.

|  | **FO1** | **FO2** | **FO3** | **FO4** | **FO5** | **FO6** | **FO7** | **FO8** | **FO9** | **FO10** |
| --- | --- | --- | --- | --- | --- | --- | --- | --- | --- | --- |
| **pH** | **3.42** ±0.01 | **3.52** ±0.01 | **3.44** ±0.01 | **3.33** ±0.01 | **3.40** ±0.01 | **4.12** ±0.01 | **3.55** ±0.01 | **3.14** ±0.01 | **3.17** ±0.01 | **3.30** ±0.01 |
| **acidity** [%] | **0.40** ±0.01 | **1.19** ±0.01 | **0.86** ±0.01 | **1.06** ±0.01 | **0.79** ±0.01 | **1.12** ±0.01 | **0.58** ±0.01 | **1.28** ±0.01 | **1.44** ±0.01 | **1.17** ±0.01 |
| **lactic acid** [mg·L^-1^] | **3974** ±155 | **8751** ±136 | **5191** ±144 | **6947** ±124 | **8874** ±199 | **2640** ±153 | **8395** ±217 | **3381** ±15 | **7557** ±273 | **4178** ±197 |
| **acetic acid** [mg·L^-1^] | **299**  ±18 | **1036** ±65 | **334** ±29 | **665**  ±35 | **530**  ±34 | **796** ±23 | **469** ±43 | **366** ±19 | **270** ±10 | **183** ±12 |
| **propionic acid** [mg·L^-1^] | **197** ±13 | **396** ±18 | **60** ±17 | **122** ±12 | **181** ±16 | **81**  ±19 | **108** ±15 | **269** ±12 | **208** ±22 | **148** ±15 |
| **butyric acid** [mg·L^-1^] | **218** ±29 | **304** ±23 | **116** ±16 | **239** ±18 | **383** ±17 | **186**  ±24 | **123**  ±18 | **317**  ±19 | **153** ±12 | **380**  ±19 |

Values are expressed as means ± standard deviations

Table S3. IQR analysis for pH and acidity of sauerkraut (FK) and fermented cucumber (FO) samples.

|  | Q1 | Q3 | IQR | lower fence | upper fence |
| --- | --- | --- | --- | --- | --- |
| pH FK | 3.49 | 3.71 | 0.22 | 3.16 | 4.03 |
| kw FK | 1.67 | 2.66 | 0.98 | 0.20 | 4.13 |
| pH FO | 3.31 | 3.50 | 0.19 | 3.02 | 3.79 |
| kw FO | 0.80 | 1.18 | 0.38 | 0.24 | 1.75 |

Table S4. IQR analysis for content of organic acid of sauerkraut (FK) and fermented cucumber (FO) samples.

| **FK** | Q1 | Q3 | IQR | lower fence | upper fence |
| --- | --- | --- | --- | --- | --- |
| lactic acid | 4179.50 | 9489.23 | 5309.73 | -3785.10 | 17453.82 |
| acetic acid | 1425.37 | 2623.52 | 1198.14 | -371.84 | 4420.73 |
| propionic acid | 190.27 | 383.58 | 193.31 | -99.70 | 673.54 |
| butyric acid | 955.96 | 3690.37 | 2734.41 | -3145.65 | 7791.98 |
| **FO** |  |  |  |  |  |
| lactic acid | 4024.72 | 8185.16 | 4160.45 | -2215.96 | 14425.84 |
| acetic acid | 308.18 | 631.46 | 323.28 | -176.74 | 1116.38 |
| propionic acid | 111.36 | 205.42 | 94.06 | -29.73 | 346.52 |
| butyric acid | 193.66 | 364.35 | 170.69 | -62.38 | 620.39 |
